# Distribution, Origins and Hazardous Effects of Polycyclic Aromatic Hydrocarbons in Topsoil Surrounding Oil Fields: A Case Study on the Loess Plateau, China

**DOI:** 10.3390/ijerph17041390

**Published:** 2020-02-21

**Authors:** Di Wang, Shilei Zhu, Lijing Wang, Qing Zhen, Fengpeng Han, Xingchang Zhang

**Affiliations:** 1 College of Natural Resources and Environment, Northwest A&F University, Yangling 712100, China; 2 State Key Laboratory of Soil Erosion and Dryland Farming on the Loess Plateau, Institute of Soil and Water Conservation, Northwest A&F University, Yangling 712100, China; 3 Institute of Soil and Water Conservation, Chinese Academy of Sciences and Ministry of Water Resources, Yangling 712100, China; 4 Xi’an Branch of Chinese Academy of Sciences, Xi’an 710043, China

**Keywords:** PAHs, distribution, sources, PMF, human health risk

## Abstract

The Loess Plateau has one of the most vulnerable ecological environments in the world, but it also contains abundant oil and gas resources that are regularly exploited, which has resulted in serious environmental problems. Therefore, it is important to analyze the polycyclic aromatic hydrocarbons (PAHs) present in the topsoil of this region. The ∑16PAHs concentrations between 1980–1999 and 2000–2019 ranged from 1134.20–15871.04 and 1010.67–18,068.80 µg kg^−1^, with average values of 5021.30 and 5662.82 µg kg^−1^. All samples displayed heavy pollution levels according to European soil quality standards. In addition, among the measured physicochemical properties, the soil organic carbon (SOC) had the greatest influence on PAHs, while soil particle size distribution had the smallest effect. Source apportionment indicated that the two main sources were petroleum source (37.57%) and vehicular traffic source (25.88%). Lastly, an assessment of the carcinogenic risks illustrated that more focus should be placed on the dermal pathway in which the human body is exposed to soil PAHs. Overall, the carcinogenic risks in different populations did not exceed 10^−4^, but there was still a potential carcinogenic risk in some age groups, especially in adult women.

## 1. Introduction

Polycyclic aromatic hydrocarbons (PAHs) are a major class of persistent organic pollutants (POPs) that are primarily generated by human activities, including the incomplete combustion of fossil fuels and biomass fuels, automobile exhaust, and oil spills [1,2,3,4,5]. They are widely distributed throughout the atmosphere, soil, water, sediment, and other environmental media. Among these, soil is the most important reservoir of PAHs, and is also a stable index used to reflect the status of environmental pollution [6]. Among the hundreds of known PAHs, the United States Environmental Protection Agency (USEPA) has identified sixteen as priority pollutants (16PAHs), seven of which are classified as carcinogenic PAHs (7PAHs) [7,8]. Due to their toxicity, mutagenicity, and carcinogenicity, the origin, distribution, and hazards of 16PAHs in soil must be analyzed [9,10,11].

China is among the major energy consumers in the world, and petroleum plays an important role in its energy production. The Loess Plateau has the largest loess coverage in the world. Because of its special geographical location, this area contains large underground oil and gas resources, making it one of the main energy bases in China [12]. The first oil well (Yanchang oil mine) on land in China was exploited in 1907, and oil exploitation in this area has developed rapidly, from more than 1 million tons at the end of the 20th century to 12.24 million tons in 2009 [13]. Although this has become a pillar of local economic development, the oil resources have been massively overexploited, resulting in severe environmental problems. Abnormal operation or maintenance will lead to the spillover and emission of petroleum compounds during petroleum extraction, storage, and transportation. Once the petroleum pollutants in the soil enter the food chain, they can pose serious health hazards to humans.

Therefore, to reveal the effects of petroleum exploitation activities on soil PAHs and to provide additional data support for local pollution remediation, 0–10 cm topsoil samples around the oil fields with varying initial exploration times (1980–1999 and 2000–2019) were sampled. The specific goals were: (1) to ascertain the concentrations and compositions of individual and total PAHs, (2) to evaluate the connection between PAHs and soil characteristics using principal component analysis, (3) to pinpoint the origin of PAHs by positive matrix factorization, and (4) to assess the possible hazards of PAHs to humans by analyzing the incremental lifetime cancer risk. The obtained results may provide great reference values for environmental protection and human health.

## 2. Materials and Methods

### 2.1. Soil Sampling and Preparation

**Figure 1:**
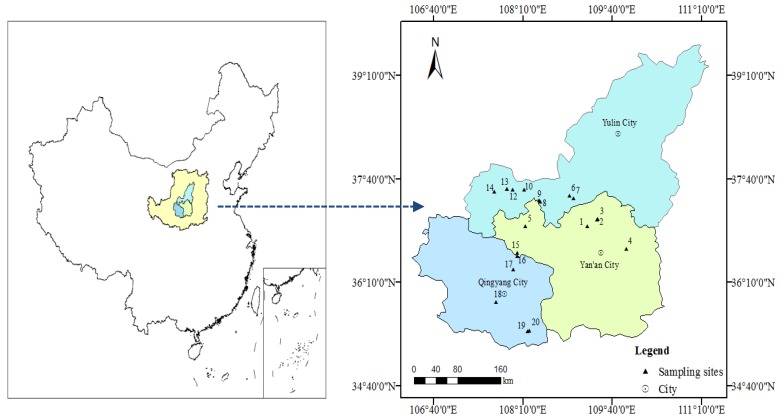
Map of the study area.

The sample collection was undertaken in July 2017 during various trips. The places for sampling belonged to the regions of Yan’an, Yulin, and Qingyang producing oils (Figure 1). All soil samples were taken near oil wells, but away from the apparent oil flows into the soil. A stainless-steel soil auger was used to collect 5 different samples at a depth of 0–10 cm soil layer and a composite sample was achieved by mixing the 5 samples. The above sampling process was repeated three times, that is, there were three composite samples at each sample point. After being taken to the laboratory, grounding of the samples was carried out followed by sieving with a sieve of stainless-steel with 60 mesh sieve and storage at 4 °C prior to analysis.

### 2.2. Analysis of Soil Physicochemical Properties

A dichromate-based oxidation method was used for measuring the soil organic carbon (SOC) content of the samples. The pH was measured in a suspension (1:2.5, soil/water) using a pH meter (Lei-ci PXSJ-216F, lei-ci, Shanghai, China). In the wet measurement mode, the soil texture was analyzed by laser diffraction using a Mastersizer 2000 (MS-2000, Malvern Panalytical, Malvern, UK).

### 2.3. PAHs Extraction and Analysis

#### 2.3.1. Reagents

Reagents mainly included anhydrous sodium sulfate (purchased from Chengdu Kelong Chemical Reagent Factory, Chengdu, China) and dichloromethane at chromatographic-grade purity (provided by Waters Company, Milford, MA, USA). The 16PAHs in a standard (made by AccuStandard Inc., New Haven, CT, USA) were Naphthalene (NAP), Acenaphthylene (ACY), Acenaphthene (ACE), Fluorene (FLU), Phenanthrene (PHE), Anthracene (ANT), Fluoranthene (FLA), Pyrene (PYR), Benzo(a)anthracene (BaA), Chrysene (CHR), Benzo(b)fluoranthene (BbF), Benzo(k)fluoranthene (BkF), Benzo(a)pyrene (BaP), Indeno(1,2,3-c,d)pyrene (InP), Dibenzo(a, h)anthracene (DBA), and Benzo(g,h,i)perylene (BgP). Extraction kits for QuEChERS were procured from Agilent Technologies Inc. (Santa Clara, CA, USA) and had the following composition: 150 mg C18, 50 mg PSA, and 900 mg Na_2_SO_4_.

#### 2.3.2. Extraction and Analysis of PAHs

The steps of analysis of PAHs mainly included dichloromethane extraction, QuEChERS reagent purification, and organic filter membrane filtration. The detailed experimental methods were described previously by Wang et al. [12]. Gas chromatography–tandem mass spectrometry (GCMS-TQ8040, Shimadzu, Kyoto, Japan) in combination with the QuEChERS method was used to determine 16PAHs. Chromatographic separation was actualized with a capillary column Rxi-5Sil Ms (30 m × 0.25 mm × 0.25 µm). He (carrier gas) flow rate was 1.0 mL min^−1^ with temperature programming as follows: increase to 50 °C with a 2 min waiting time followed by an increase at a rate of 20 °C min^−1^ up to 250 °C with a 3 min waiting time, and a final increase to 300 °C at a heating rate of 5 °C min^−1^ with a 5 min waiting time. Identification of PAHs was implemented by comparison of retention time and ion characteristics with standards (Supplementary Table S1).

#### 2.3.3. Detection of PAHs

The external standard method having seven points was used for 16PAHs described by a linear equation having R^2^ > 0.996 (Supplementary Table S2). The limit of detection (LOD) was found to be thrice to that of the standard deviation of the blank. The LOD of PAHs was in the range of 0.02–0.80 μg kg^−1^. Any concentrations below the LOD were defined as non-detected (N.D.). The 16PAHs had an average recovery of 65–119% with 0.5–9.5% being the range of relative standard deviation (RSD). The recoveries and RSDs were found to be in accordance with the experimental standard (Supplementary Table S3).

### 2.4. Data Analysis

#### 2.4.1. Source Apportionment

PMF (Positive Matrix Factorization) is an analysis tool of the source apportionment of pollutants recommended by USEPA [14]. The tool is applicable for different environmental media such as soil/sediment, water, and air samples. The PMF model is aimed at calculating the lowest objective function *Q* for ‘*m*’ number of sources containing ‘*n*’ samples in terms of the residuals (*e_ij_*) and uncertainty (*u_ij_*):

(1) $$Q=\overset{n}{\underset{i=1}{\sum }}\overset{m}{\underset{j=1}{\sum }}{\left(\frac{x_{ij}-\sum_{k=1}^{p}g_{ik}f_{kj}}{u_{ij}}\right)}^{2}$$

where, for measurement of a source ‘*j*’ in sample ‘*i*’ in the presence of ‘*p*’ number of factors, *u_ij_* is the uncertainty estimate, the contribution of each factor towards the individual sample is *g_ik_*, and the profile of species in each source is given by *f_kj_*.

For each sample, the uncertainties were a combination of uncertainty in measurement and detection limits of the method (*MDL*). For samples having a concentration equal to or below *MDL*, the uncertainty *U_nc_* was taken as a fixed fraction of the *MDL*:

(2) $$U_{nc}=\frac{5}{6}MDL$$

but for higher sample concentrations (>*MDL*), *U_nc_* was based on our provided fraction of the concentration and *MDL*:

(3) $$U_{nc}=\sqrt{{\left(Error\;fraction\right)}^{2}+{\left(0.5\times MDL\right)}^{2}}$$

Starting from different points and choosing 3–5 variables, the PMF model was run after selecting a random seed mode having 33 starting points chosen randomly. The number of factors corresponding to PMF was determined after comparison of the values *Q* true and *Q* robust, where *Q* true is a measure of closeness of the input data to the fit parameter and *Q* robust is achieved after excluding the outliers [15]. The *Q* robust value (2165.9) calculated by PMF was close to the *Q* true value (2167.2). Therefore, the analytical results of PMF were reasonable. Hence, optimal solution based on four factors indicated a strong relation between observed and predicted PAH concentrations (r^2^ > 0.90, *p* < 0.01). Three different types of PAHs were identified by the PMF model: strong (FLU, ANT, FLA, PYR, and BaA), weak (NAP, ACE, PHE, CHR, BbF, BkF, BaP, InP, DBA, and BgP), and bad species (ACY).

#### 2.4.2. Health Risk Assessment

According to the appraisal of health risk, different PAHs have different toxicities [2]. In this work, the direct exposure risk of PAHs towards cancer was investigated using the incremental lifetime cancer risk (ILCR). This model based on ILCR was used in combination with a toxic equivalent method based on toxicity equivalency factors (TEFs). High carcinogenicity of BaP influenced its choice as a reference compound in the TEF method [16]. The *ILCRs* included ingestion, dermal contact, and inhalation [17]. They were calculated through the following formula:

(4) $$CS=\sum \left({PAH}_{i}\times {TEF}_{i}\right)$$

(5) $${ILCR}_{ingestion}=\frac{CS\times \left({CSF}_{ingestion}\times \sqrt[{3}]{\frac{BW}{70}}\right)\times IngR\times EF\times ED}{BW\times AT\times 10^{6}}$$

(6) $${ILCR}_{dermal}=\frac{CS\times \left({CSF}_{dermal}\times \sqrt[{3}]{\frac{BW}{70}}\right)\times SA\times AF\times ABS\times EF\times ED}{BW\times AT\times 10^{6}}$$

(7) $${ILCR}_{inhalation}=\frac{CS\times \left({CSF}_{inhalation}\times \sqrt[{3}]{\frac{BW}{70}}\right)\times InhR\times EF\times ED}{BW\times AT\times PEF}$$

(8) $$ILCRs=\sum \left({ILCR}_{ingestion}+{ILCR}_{dermal}+{ILCR}_{inhalation}\right)$$

where *PAH_i_* denotes the average soil concentration of a single PAH (mg kg^−1^), *TEF_i_* is the toxic equivalency factor for an individual PAH in relation to BaP, and *CS* denotes the summation of converted PAH concentrations based on TEFs. CSF denotes the carcinogenic slope factor (mg kg^−1^ day^−1^)^−1^, where *CSF_ingestion_*, *CSF_dermal_*, and *CSF_inhalation_* were assigned the values of 7.3, 25, and 3.85 (mg kg^−1^ day^−1^)^−1^, respectively [18]. *BW* refers to the body weight (kg), *EF* refers to the exposure frequency (d year^−1^), *ED* is the duration of exposure (year), and *InhR* and *IngR* denote the rate of inhalation (m^3^ d^−1^) and the rate of soil ingestion (mg d^−1^), respectively. *SA* corresponds to the surface area of dermal exposure (cm^2^ day^−1^), the dermal adherence factor is referred to *AF* (mg cm^−2^), *AT* denotes the average life span (day), *ABS* is the dermal adsorption factor, and *PEF* corresponds to the particle emission factor (m^3^ kg^−1^). People were divided in terms of age and gender in six groups, such as: (1) adult male (18–70 years age), (2) adult female (18–70 years age), (3) adolescent male (11–17 years age), (4) adolescent female (11–17 years age), (5) male child (2–10 years age), and (6) female child (2–10 years age) [19]. The corresponding parameter values are given in Supplementary Table S4.

#### 2.4.3. Statistical Analysis

SPSS 25.0 software (IBM Corporation, Armonk, NY, USA) was used for statistical treatment of data. The sampling diagram was drawn by Arcgis 10.2 software (ESRI Inc., Redlands, California, USA), whereas other figures were drawn by Origin 9.0 software (OriginLab, Northampton, MA, USA). The inter relation among individual PAH and soil physicochemical properties was by Principal Component Analysis (PCA) with Canoco 5.0. USEPA PMF 5.0 (ExoAnalytics Inc., Washington, DC, USA) was used to identify and analyze PAH sources.

## 3. Results and Discussion

### 3.1. Concentrations and Compositions of PAHs

The concentration profiles of PAHs around oil wells of different initial production time are shown in Table 1. The ∑16PAHs concentrations between 1980–1999 and 2000–2019 ranged from 1134.20–15,871.04 and 1010.67–18,068.80 µg kg^−1^, with a mean of 5021.30 and 5662.82 µg kg^−1^, respectively. The summation of seven carcinogenic PAHs (∑7PAHs) concentrations in the corresponding time ranged from 370.96–4214.10 and 223.96–4642.40, with an average of 1658.93 and 1877.19 µg kg^−1^, respectively. According to the structural characteristics, 16PAHs can be divided into two types: LMWPAHs (2–3 rings) and HMWPAHs (≥4 rings). The LMWPAHs concentrations between 1980–1999 and 2000–2019 varied from 449.01–10,008.86 µg kg^−1^ (mean 2775.74 µg kg^−1^) and 307.07–11,805.44 µg kg^−1^ (mean 2890.40 µg kg^−1^), respectively. While HMWPAHs concentrations in the corresponding time varied from 527.78–5862.18 (mean 2245.56 µg kg^−1^) and 544.86–6263.36 µg kg^−1^ (mean 2772.41 µg kg^−1^), respectively. In general, the concentration of HMWPAHs is generally higher than those in LMWPAHs because of the more significant degradation profile of LMWPAHs [20]. Moreover, the relatively high resistance of HMWPAHs to degradation contributes to their accumulation in soil [21]. It is evident from Table 1 that the pollution of soil PAHs in 2000–2019 is more serious than that in 1980–1999. One reason is that the utilization rate of oil wells is low in the first two decades (some oil wells have been abandoned), and the pollution degree of soil PAHs has been alleviated after a long period of natural degradation and remediation. The other reason is that the utilization rate and oil recovery rate of newer oil wells are relatively high, so the leakage of oil and the emission of exhaust gas from oil production vehicles will aggravate the pollution of soil PAHs in the process of oil exploitation.

**Figure 2:**
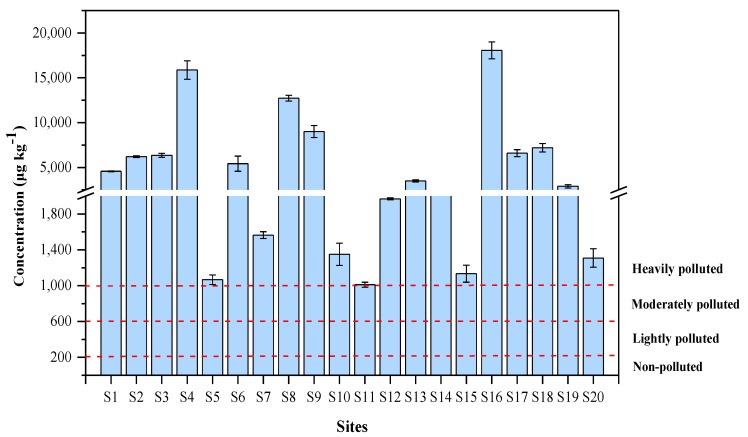
Concentrations of PAHs in soil samples.

The soil PAHs pollution was separated into four grades with reference to the soil quality standard proposed by Maliszewska-Kordybach [22]. The total concentration of 16PAHs below 200 µg kg^−1^ indicates non-polluted, with lightly polluted being indicated by a concentration of 200–600 µg kg^−1^, and a concentration of 600–1000 µg kg^−1^ representing moderately polluted. When the concentration exceeds 1000 µg kg^−1^, the area is heavily polluted. Compared to the above classification standards, all sites were heavily contaminated (Figure 2).

**Figure 3:**
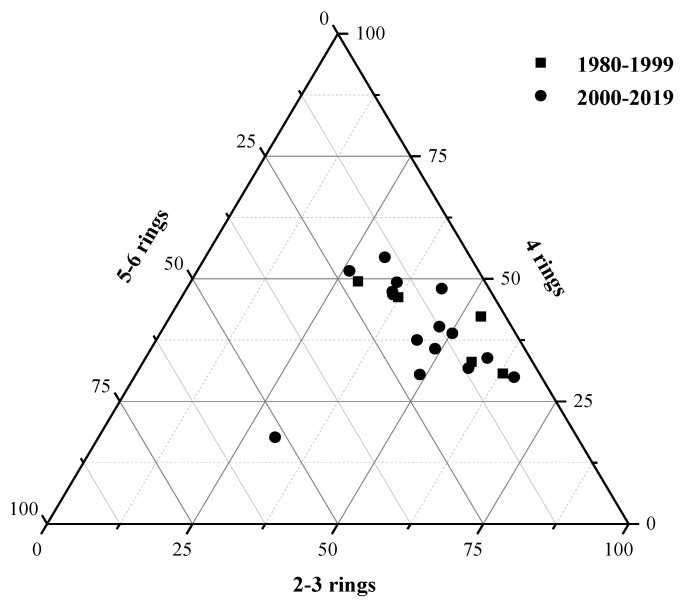
Triangular diagram of percentage concentrations for 16PAHs in soil samples.

According to the number of aromatic rings, the 16PAHs were classified into three different groups, one with 2–3 rings, another with 4 rings, and last one with 5–6 rings [23]. The compositions of soil PAHs around oil wells with different production times are shown in Figure 3. In 1980–1999, the proportion of PAHs having 2–3 rings, 4 rings, and 5–6 rings in this region was 28.71−63.06% (mean 47.80%), 30.67–49.48% (mean 40.35%), and 4.24–21.81% (mean 11.86%), respectively. Similar results were found in 2000–2019, the highest ratio value of soil PAHs was found in 2–3 rings (26.20–65.33%, mean 43.97%), followed by 4 rings (17.67–54.38%, mean 39.57%), and 5–6 rings (4.72–51.95%, mean 16.47%). It can be found that the proportion of LMWPAHs is large, which are mainly related to combustion products of pyrogenic processes at low temperatures [24]. However, HMWPAHs are more likely to accumulate in soil because of their low volatility, and their sources are different from those of LMWPAHs. Generally, LMWPAHs are produced by substances like petroleum and its derivatives, while HMWPAHs mainly come from the combustion of coal, organic compounds, and higher plants [25]. The specific sources of PAHs will be discussed below.

### 3.2. Relationships between PAHs and Environmental Factors

**Figure 4:**
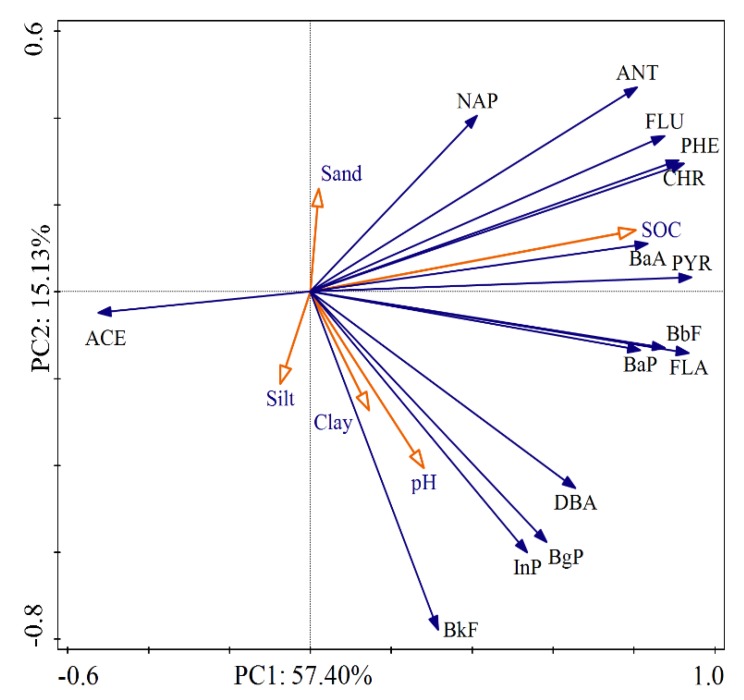
Principal component analysis (PCA) of the correlation between PAHs and soil physicochemical properties. All the PAHs abbreviations used are explained in Supplementary Table S1.

As shown in Figure 4, PCA provided a single two-dimensional model, which would explain 72.53% of the variance in the data. It can be found that there is a high correlation between individual PAHs. Reflection of calculated Pearson correlation coefficients for the individual PAHs showed interrelation of most variables at level 0.01 (Supplementary Table S5).

Commonly, soil properties such as pH, SOC, and texture, are important factors affecting the concentrations of PAHs [26]. In our study, the vector magnitude of environmental factors indicated that SOC was the most significant factor. The soils with high SOC had a strong adsorption ability to PAHs, which affected the distribution and migration of PAHs in the environment. Poor correlation existed between pH and PAHs, indicating that pH was a non-critical parameter affecting PAHs. For soil particle size distribution, we found that the influence of sand, silt, and clay on the distribution of PAHs was basically the same. The difference was that most individual PAHs showed positive correlations with soil clay, whereas negative correlations with soil sand, suggesting that PAHs were more likely to accumulate in fine soil particles. The reason is that small particles revealed stronger affinities of PAHs than larger particles due to the higher surface area of smaller particles.

### 3.3. Source Analysis of PAHs

**Figure 5:**
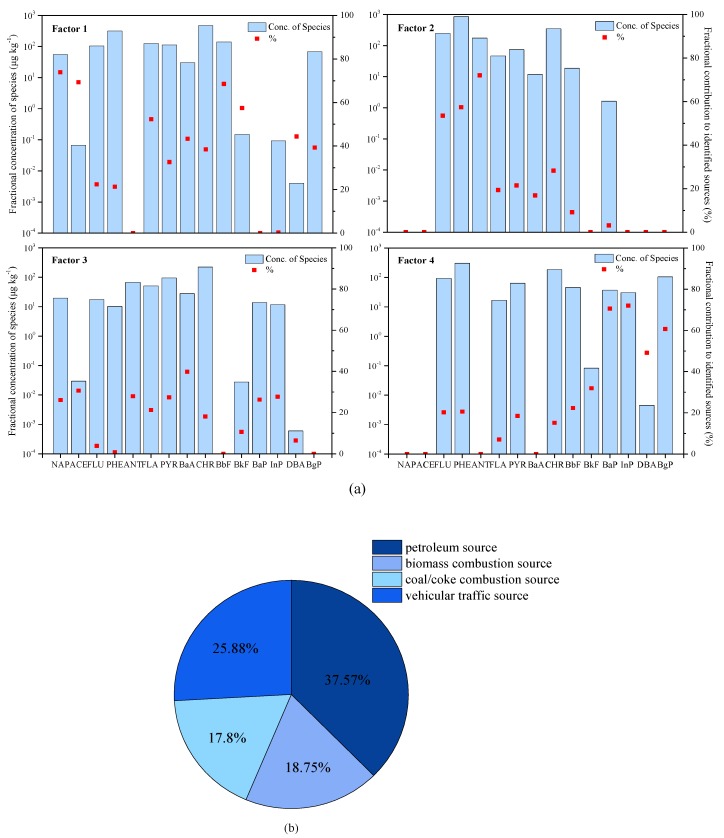
Results of the Positive Matrix Factorization (PMF) model: (**a**) Source profiles of each PMF factor and (**b**) percentage source contributions of each factor to total PAHs. All the PAHs abbreviations used are explained in Supplementary Table S1.

As this study was carried out using samples from locations around oil wells, we can relate the main sources of 16PAHs with oil exploitation and human-related activities. But, it is also required to take into account other sources. For further identification of PAHs sources, modeling of the data was carried out by PMF [27]. Figure 5a presents the average contributions of PAHs species towards four PMF factors.

A major portion (37.57%) of the total measured PAHs was due to the factor 1, which was loaded predominately by NAP and ACE. Among all the different PAHs, NAP was the heaviest and was usually used as an indicator for the leakage of petroleum substances [28,29,30], whereas ACE was mainly from the combustion of petroleum-based fuel at lower temperatures. Earlier investigations have indicated that origins of LMWPAHs are petroleum products related to petrogenic sources [6,31,32]. Therefore, factor 1 was marked as related to petroleum sources. ANT, PHE, and FLU, which are believed to be the tracers of wood/grass burning, dominated factor 2, which accounted for 18.75% of the total PAHs [33,34,35,36,37]. Hence, factor 2 was identified as originating from burning of biomass. Factor 3 explained 17.80% of the total PAHs and heavily loaded on BaA, followed by ACE, ANT, PYR, NAP, InP, and BaP. Among them, BaA was not only the characteristic indicator of natural gas, but also the product of coal combustion [38,39]. Other PAHs were related to the release from burning of coal and coke combustion [40,41]. Thus, factor 3 was related to the combustion of coal/coke. Factor 4 explained 25.88% variance of the data and was high loaded predominately by molecular weight PAHs with a high loading on InP and BaP and moderately by BgP and DBA. InP and BgP were characteristic indicators of diesel and gasoline combustion, respectively [42]. Meanwhile, particulate matters of gasoline vehicle exhaust showed high emissions of InP and DBA [43,44]. Consequently, factor 4 was related to the source of vehicular traffic.

The PMF results showed the following order for relative contributions to the total soil PAHs burden: petroleum source (37.57%) > vehicular traffic source (25.88%) > biomass combustion source (18.75%) > coal/coke combustion source (17.80%) (Figure 5b). The rationalization proposal is to pay attention to the leakage and sprinkling of petroleum in the process of petroleum exploitation. Moreover, car drivers are advised to replace gasoline and diesel with cleaner fuels, or to use electric vehicles, which can reduce the burden of PAHs in the soil environment.

### 3.4. Assessment of Health Risk

The results of lifetime cancer risk levels are given in Table 2. Normally, values of *ILCRs* indicate the magnitude of risk with negligible risks, potential health risks, and higher risks being denoted by the values of 10^−6^, between 10^−6^ to 10^−4^, and greater than 10^−4^, respectively [45]. In the current study, it is clear that all soil samples had the same level of cancer risk (10^−12^–10^−10^) through inhalation in all soil samples, indicating that there is no cancer risk during different periods. By contrast, the levels of carcinogenic risk to the human body are large through ingestion and dermal contact pathways.

For the ingestion pathway, the average *ILCR_ingestion_* (1980–1999) for male and female were estimated to be 8.54 × 10^−7^, 5.09 × 10^−7^, and 9.26 × 10^−7^, and 8.78 × 10^−7^, 5.26 × 10^−7^, and 1.01 × 10^−6^, for children, adolescents, adults respectively, suggesting that adults were most sensitive to PAHs contamination. In addition, adults have the highest risk (mean value: 1.65 × 10^−6^–1.79 × 10^−6^) of exposure to soil PAHs through the dermal pathway, followed by adolescents (mean value: 1.27 × 10^−6^–1.31 × 10^−6^) and children (mean value: 1.06 × 10^−6^–1.09 × 10^−6^). Accurately, the carcinogenic risk of male and female in children and adolescents were basically the same, while in adults, the carcinogenic risk in women was higher than that in men. The higher inhalation rate with larger dermal exposure area along with exposure duration for adults increases carcinogenic risk. Moreover, the weight difference between men and women is responsible for higher carcinogenic risk in women. These results indicated that the dermal exposure of the human body towards soil PAHs is the most important in this region, causing great concern to us. In 2000–2019, the toxin effect of PAHs from soil to all the groups through direct ingestion, dermal contact, and inhalation was basically the same as that in 1980–1999. However, the mean value of *ILCRs* in 2000–2019 is higher than that in 1980–1999 owing to the rapid demand for petroleum during the recent two decades, resulting in the soil being seriously polluted. So, administrators in petroleum production areas on the Loess Plateau need to take some steps to reduce the risks posed by PAHs.

## 4. Conclusions

Understanding the distribution, origins, and potential health hazards of 16PAHs in soil around oil wells on the Loess Plateau is critical to appropriately manage PAHs in the soil matrice. The pollution of soil PAHs in 2000–2019 is more serious than that in 1980–1999. Among the measured PAHs, the ratio of soil PAHs decreased in the following order with respect to the number of rings: 2–3 > 4 > 5–6. SOC was the most significant factor, followed by pH and soil particle size distribution. Four different sources were identified by PMF. Source apportionment revealed that origins of PAHs were mainly from petroleum and vehicular traffic. Biomass and coal/coke combustion also contribute to the PAHs recorded in this area. The ILCRs results indicated that the exposure pathway of dermal contact was more noteworthy than that of ingestion and inhalation. Moreover, the health risk of adults is higher than that of adolescents and children.

## Figures and Tables

**Table 1:** Concentration profiles of PAHs in soil samples (μg kg^−1^).

Compounds	1980–1999			2000–2019		
	Mean	Median	Range	Mean	Median	Range
NAP	125.06	105.08	64.46–196.55	106.54	93.35	66.13–173.61
ACY	N.D.	N.D.	N.D.	N.D.	N.D.	N.D.
ACE	92.76	67.08	N.D.–157.98	117.35	129.18	N.D.–148.78
FLU	447.80	160.88	78.05–1665.20	471.17	178.38	44.48–2383.70
PHE	1857.66	770.92	200.39–6836.28	2027.13	1709.03	127.81–8052.24
ANT	361.96	56.82	N.D.–1317.29	247.27	123.33	N.D.–1238.50
FLA	167.70	101.15	48.14–517.14	301.39	291.39	23.09–867.22
PYR	274.51	146.45	82.95–872.94	366.73	358.11	34.62–818.16
BaA	58.72	35.56	N.D.–136.92	82.11	73.25	4.20–185.51
CHR	1287.65	493.29	296.07–3340.34	1357.79	1226.35	116.65–3796.53
BbF	189.04	150.16	48.07–449.25	244.37	185.32	27.08–572.11
BkF	18.04	18.55	N.D.–21.09	35.50	28.15	N.D.–67.05
BaP	100.91	82.55	N.D.–214.56	96.00	73.11	N.D.–225.40
InP	44.69	56.53	N.D.–71.32	74.97	58.65	N.D.–151.52
DBA	42.28	42.28	N.D.–55.69	65.33	67.69	N.D.–102.03
BgP	180.53	185.63	N.D.–294.00	227.11	217.56	65.30–433.26
∑16PAHs	5021.30	1968.79	1134.20–15,871.04	5662.82	5418.25	1010.67–18,068.80
∑7PAHs	1658.93	753.88	370.96–4214.10	1877.19	1950.37	223.96–4642.40
∑LMWPAHs	2775.74	1112.14	449.01–10,008.86	2890.40	2430.40	307.07–11,805.44
∑HMWPAHs	2245.56	1114.75	527.78–5862.18	2772.41	2614.66	544.86–6263.36

∑16PAHs—summation of concentrations of sixteen PAHs. ∑7PAHs—summation of concentrations of seven carcinogenic PAHs including BaA, CHR, BbF, BkF, BaP, DBA, and InP. ∑LMWPAHs—the concentration sum of low molecular weight PAHs (2–3 rings). ∑HMWPAHs—the total concentrations of high molecular weight PAHs (≥4 rings). All the other abbreviations used are explained in Supplementary Table S1. N.D.—Not Detected.

**Table 2:** The *ILCRs* of people for different exposure pathways.

Exposure Pathway			1980–1999				2000–2019			
			*ILCR_ingestion_*	*ILCR_dermal_*	*ILCR_inhalation_*	*ILCRs*	*ILCR_ingestion_*	*ILCR_dermal_*	*ILCR_inhalation_*	*ILCRs*
Child	Male	Min	2.91 × 10^−7^	3.63 × 10^−7^	6.15 × 10^−12^	6.54 × 10^−7^	1.33 × 10^−7^	1.66 × 10^−7^	2.82 × 10^−12^	3.00 × 10^−7^
		Max	1.74 × 10^−6^	2.17 × 10^−6^	3.67 × 10^−11^	3.91 × 10^−6^	2.15 × 10^−6^	2.68 × 10^−6^	4.55 × 10^−11^	4.83 × 10^−6^
		Mean	8.54 × 10^−7^	1.06 × 10^−6^	1.81 × 10^−11^	1.92 × 10^−6^	9.91 × 10^−7^	1.24 × 10^−6^	2.09 × 10^−11^	2.23 × 10^−6^
	Female	Min	2.99 × 10^−7^	3.73 × 10^−7^	6.33 × 10^−12^	6.72 × 10^−7^	1.37 × 10^−7^	1.71 × 10^−7^	2.90 × 10^−12^	3.08 × 10^−7^
		Max	1.79 × 10^−6^	2.23 × 10^−6^	3.78 × 10^−11^	4.02 × 10^−6^	2.21 × 10^−6^	2.76 × 10^−6^	4.67 × 10^−11^	4.97 × 10^−6^
		Mean	8.78 × 10^−7^	1.09 × 10^−6^	1.86 × 10^−11^	1.97 × 10^−6^	1.02 × 10^−6^	1.27 × 10^−6^	2.15 × 10^−11^	2.29 × 10^−6^
Adolescent	Male	Min	1.74 × 10^−7^	4.33 × 10^−7^	1.19 × 10^−11^	6.06 × 10^−7^	7.95 × 10^−8^	1.98 × 10^−7^	5.46 × 10^−12^	2.78 × 10^−7^
		Max	1.04 × 10^−6^	2.58 × 10^−6^	7.11 × 10^−11^	3.62 × 10^−6^	1.28 × 10^−6^	3.20 × 10^−6^	8.80 × 10^−11^	4.48 × 10^−6^
		Mean	5.09 × 10^−7^	1.27 × 10^−6^	3.50 × 10^−11^	1.78 × 10^−6^	5.91 × 10^−7^	1.47 × 10^−6^	4.05 × 10^−11^	2.06 × 10^−6^
	Female	Min	1.79 × 10^−7^	4.47 × 10^−7^	1.23 × 10^−11^	6.27 × 10^−7^	8.22 × 10^−8^	2.05 × 10^−7^	5.64 × 10^−12^	2.87 × 10^−7^
		Max	1.07 × 10^−6^	2.67 × 10^−6^	7.35 × 10^−11^	3.74 × 10^−6^	1.33 × 10^−6^	3.30 × 10^−6^	9.10 × 10^−11^	4.63 × 10^−6^
		Mean	5.26 × 10^−7^	1.31 × 10^−6^	3.61 × 10^−11^	1.84 × 10^−6^	6.11 × 10^−7^	1.52 × 10^−6^	4.19 × 10^−11^	2.13 × 10^−6^
Adult	Male	Min	3.16 × 10^−7^	5.61 × 10^−7^	2.14 × 10^−11^	8.77 × 10^−7^	1.45 × 10^−7^	2.57 × 10^−7^	9.81 × 10^−12^	4.02 × 10^−7^
		Max	1.89 × 10^−6^	3.35 × 10^−6^	1.28 × 10^−10^	5.24 × 10^−6^	2.33 × 10^−6^	4.14 × 10^−6^	1.58 × 10^−10^	6.48 × 10^−6^
		Mean	9.26 × 10^−7^	1.65 × 10^−6^	6.29 × 10^−11^	2.57 × 10^−6^	1.07 × 10^−6^	1.91 × 10^−6^	7.29 × 10^−11^	2.98 × 10^−6^
	Female	Min	3.43 × 10^−7^	6.10 × 10^−7^	2.33 × 10^−11^	9.53 × 10^−7^	1.57 × 10^−7^	2.79 × 10^−7^	1.07 × 10^−11^	4.37 × 10^−7^
		Max	2.05 × 10^−6^	3.64 × 10^−6^	1.39 × 10^−10^	5.69 × 10^−6^	2.54 × 10^−6^	4.50 × 10^−6^	1.72 × 10^−10^	7.04 × 10^−6^
		Mean	1.01 × 10^−6^	1.79 × 10^−6^	6.84 × 10^−11^	2.80 × 10^−6^	1.17 × 10^−6^	2.08 × 10^−6^	7.93 × 10^−11^	3.24 × 10^−6^

## Supplementary Materials

The following are available online at https://www.mdpi.com/1660-4601/17/4/1390/s1, Table S1: Retention time and ion characteristics of the selected PAHs in the MRM mode; Table S2: Equations of calibration curve of 16PAHs; Table S3: Performance and validation of the analytical method (*n* = 6); Table S4: Parameters used in the incremental lifetime cancer risk assessment; Table S5: Correlation analysis between PAHs and environmental factors.
