# Supplementary material for: Distribution, Origins and Hazardous Effects of Polycyclic Aromatic Hydrocarbons in Topsoil Surrounding Oil Fields: A Case Study on the Loess Plateau, China
Source: Int J Environ Res Public Health. 2020 Feb 21;17(4):1390. doi: 10.3390/ijerph17041390 (PMC7068613; doi:10.3390/ijerph17041390)
Supplement: Supplementary file 1 [file ijerph-17-01390-s001.pdf]

Table S1. Retention time and ion characteristics of the selected PAHs in the MRM mode.

Compounds	CAS Number	RetentionTime	Quantitative Ion Pair	Qualitative Ion Pair
NAP	91-20-3	7.450	128.1 > 102.1(20)	128.1 > 78.0(20)
ACY	208-96-8	9.420	152.1 > 150.1(28)	152.1 > 126.1(28)
ACE	83-32-9	9.630	153.1 > 151.1(28)	153.1 > 127.1(28)
FLU	86-73-7	10.265	165.1 > 163.1(28)	165.1 > 139.1(28)
PHE	85-01-8	11.455	178.1 > 176.1(28)	178.1 > 152.1(20)
ANT	120-12-7	11.515	178.1 > 176.1(28)	178.1 > 152.1(20)
FLA	206-44-0	13.055	202.1 > 200.1(30)	200.1 > 198.1(30)
PYR	129-00-0	13.435	202.1 > 200.1(30)	200.1 > 198.1(30)
BaA	56-55-3	16.450	228.1 > 226.1(32)	226.1 > 224.1(32)
CHR	218-01-9	16.575	228.1 > 226.1(32)	226.1 > 224.1(32)
BbF	205-99-2	20.110	252.1 > 250.1(36)	250.1 > 248.1(36)
BkF	207-08-9	20.205	252.1 > 250.1(36)	250.1 > 248.1(36)
BaP	50-32-8	21.230	252.1 > 250.1(36)	250.1 > 248.1(36)
InP	193-39-5	25.015	276.1 > 274.1(36)	274.1 > 272.1(36)
DBA	53-70-3	25.150	276.1 > 274.1(36)	274.1 > 272.1(36)
BgP	191-24-2	25.865	276.1 > 274.1(36)	274.1 > 272.1(36)

Naphthalene (NAP), Acenaphthylene (ACY), Acenaphthene (ACE), Fluorene (FLU), Phenanthrene (PHE), Anthracene (ANT), Fluoranthene (FLA), Pyrene (PYR), Benzo(a)anthracene (BaA), Chrysene (CHR), Benzo(b)fluoranthene (BbF), Benzo(k)fluoranthene (BkF), Benzo(a)pyrene (BaP), Indeno(1,2,3-c,d)pyrene (InP), Dibenzo(a, h)anthracene (DBA), Benzo(g,h,i)perylene (BgP).

Table S2. Equations of calibration curve of 16PAHs.

Compounds	Correlation Curve Equation	Correlation Coefficient
NAP	y = 63,176x+519.17	0.9994
ACY	y = 28,879x+73.85	0.9995
ACE	y = 21,942x+119.61	0.9994
FLU	y = 34,547x+212.68	0.9994
PHE	y = 57,772x+616.73	0.9993
ANT	y = 56,434x+121.88	0.9995
FLA	y = 91,879x+251.04	0.9993
PYR	y = 91,977x+317.27	0.9993
BaA	y = 116,050x-168.32	0.9992
CHR	y = 118,325x+3.03	0.9994
BbF	y = 77,885x-830.47	0.9968
BkF	y = 72,726x-343.48	0.9995
BaP	y = 61,367x-316.60	0.9992
InP	y = 33,770x+13.98	0.9994
DBA	y = 44,048x-119.35	0.9993
BgP	y = 32,355x-29.65	0.9980

All the abbreviations used were explained in Table S1.

Table S3. Performance and validation of the analytical method (*n* = 6).

Compounds	200 μg kg^{-1}		1000 μg kg^{-1}		Detection Limit (μg kg^{-1})
	Recovery (%)	RSD (%)	Recovery (%)	RSD (%)	
NAP	110.2	5.66	118.3	1.65	0.02
ACY	109.2	0.88	117.2	0.50	0.80
ACE	105.4	5.32	119.0	3.46	0.60
FLU	108.2	4.11	112.1	0.83	0.12
PHE	106.4	3.12	109.4	6.65	0.04
ANT	90.2	7.90	94.3	6.38	0.16
FLA	90.1	6.13	109.1	1.10	0.12
PYR	106.3	7.52	110.1	5.93	0.16
BaA	103.1	4.22	95.2	2.78	0.18
CHR	109.1	3.19	96.2	3.53	0.08
BbF	88.5	8.21	98.1	9.09	0.16
BkF	95.1	5.31	93.2	7.55	0.20
BaP	60.2	7.35	65.2	8.90	0.20
InP	89.3	5.12	93.0	6.33	0.06
DBA	88.1	9.08	94.1	3.93	0.06
BgP	92.2	8.90	80.4	9.50	0.10

All the abbreviations used were explained in Table S1.

Table S4. Parameters used in the incremental lifetime cancer risk assessment.

Exposure variable	Unit	Child		Adolescent		Adult	
		Male	Female	Male	Female	Male	Female
Body weight (BW)	kg	17.2	16.5	47.1	44.8	60.2	53.1
Exposure frequency (EF)	$day \cdot year^{-1}$	350	350	350	350	350	350
Exposure duration (ED)	year	6	6	14	14	30	30
Inhalation rate (InhR)	$m^3 \cdot day^{-1}$	10.9	10.9	17.7	17.7	17.5	17.5
Soil ingestion rate (IngR)	$mg \cdot day^{-1}$	200	200	100	100	100	100
Dermal exposure area (SA)	$cm^2 \cdot day^{-1}$	2800	2800	2800	2800	5700	5700
Dermal adherence factor (AF)	$mg \cdot cm^{-2}$	0.2	0.2	0.2	0.2	0.07	0.07
Averaging life span (AT)	day	70 × 365 = 25,550	70 × 365 = 25,550	70 × 365 = 25,550	70 × 365 = 25,550	70 × 365 = 25,550	70 × 365 = 25,550
Particle emission factor (PEF)	$m^3 \cdot kg^{-1}$	1.36×10^9	1.36×10^9	1.36×10^9	1.36×10^9	1.36×10^9	1.36×10^9
Dermal adsorption factor (ABS)	Unitless	0.13	0.13	0.13	0.13	0.13	0.13
Carcinogenic slope factor (CSF) Ingestion	$(mg \cdot kg^{-1} \cdot day^{-1})^{-1}$	7.3	7.3	7.3	7.3	7.3	7.3
Carcinogenic slope factor (CSF) Dermal	$(mg \cdot kg^{-1} \cdot day^{-1})^{-1}$	25	25	25	25	25	25
Carcinogenic slope factor (CSF) Inhalation	$(mg \cdot kg^{-1} \cdot day^{-1})^{-1}$	3.85	3.85	3.85	3.85	3.85	3.85

Table S5. Correlation analysis between PAHs and environmental factors.

Types	pH	SOC	Sand	Silt	Clay	NAP	ACE	FLU	PHE	ANT	FLA	PYR	BaA	CHR	BbF	BkF	BaP	InP	DBA	BgP
pH	1																			
SOC	0.128	1																		
Sand	−0.067	0.118	1																	
Silt	0.004	−0.105	−0.986 **	1																
Clay	0.245	−0.138	−0.866 **	0.772 **	1															
NAP	0.021	0.443	0.410	−0.395	−0.382	1														
ACE	0.115	−0.290	−0.028	0.068	−0.098	−0.203	1													
FLU	0.127	0.683 **	0.073	−0.148	0.166	0.375	−0.458 *	1												
PHE	0.146	0.764 **	0.160	−0.209	0.018	0.483 *	−0.429	0.949 **	1											
ANT	−0.059	0.659 **	0.050	−0.101	0.113	0.375	−0.444	0.928 **	0.890 **	1										
FLA	0.372	0.703 **	−0.124	0.069	0.271	0.180	−0.382	0.801 **	0.844 **	0.690 **	1									
PYR	0.194	0.818 **	−0.077	0.049	0.147	0.273	−0.437	0.808 **	0.839 **	0.830 **	0.898 **	1								
BaA	0.188	0.671 **	−0.138	0.113	0.187	0.221	−0.301	0.728 **	0.744 **	0.748 **	0.812 **	0.887 **	1							
CHR	0.107	0.885 **	0.173	−0.200	−0.062	0.584 **	−0.471 *	0.883 **	0.930 **	0.852 **	0.789 **	0.876 **	0.788 **	1						
BbF	0.403	0.603 **	−0.089	0.010	0.311	0.290	−0.410	0.768 **	0.762 **	0.626 **	0.911 **	0.765 **	0.671 **	0.728 **	1					
BkF	0.288	0.018	−0.258	0.183	0.438	−0.326	−0.089	0.115	0.117	0.006	0.450 *	0.258	0.105	0.022	0.462 *	1				
BaP	0.352	0.772 **	0.096	−0.131	0.026	0.385	−0.473 *	0.660 **	0.652 **	0.517 *	0.729 **	0.742 **	0.533 *	0.757 **	0.728 **	0.302	1			
InP	0.438	0.441	−0.135	0.110	0.187	0.060	−0.246	0.169	0.204	0.127	0.509 *	0.559 *	0.471 *	0.338	0.441	0.404	0.608 **	1		
DBA	0.528 *	0.438	−0.087	0.074	0.111	0.261	−0.328	0.298	0.469 *	0.240	0.672 **	0.601 **	0.585 **	0.470 *	0.566 **	0.371	0.454 *	0.696 **	1	
BgP	0.285	0.450 *	0.173	−0.224	0.012	0.246	−0.299	0.316	0.420	0.174	0.543 *	0.425	0.278	0.450 *	0.535 *	0.680 **	0.573 **	0.539 *	0.595 **	1

Note: * Correlation is significant at $p < 0.05$ (two-tailed); ** Correlation is significant at $p < 0.01$ (two-tailed). All the PAHs abbreviations used were explained in Table S1.
